# Determinants of first trimester attendance at antenatal care clinics in the Amazon region of Peru: A case-control study

**DOI:** 10.1371/journal.pone.0171136

**Published:** 2017-02-16

**Authors:** Nora Moore, Brittany Blouin, Hugo Razuri, Martin Casapia, Theresa W. Gyorkos

**Affiliations:** 1 Department of Epidemiology, Biostatistics and Occupational Health, McGill University, Montréal, Canada; 2 Division of Clinical Epidemiology, McGill University Health Centre, Montréal, Canada; 3 Asociación Civil Selva Amazónica, Iquitos, Peru; Iranian Institute for Health Sciences Research, ISLAMIC REPUBLIC OF IRAN

## Abstract

**Objective:**

To identify determinants which influence the timing of the first antenatal care (ANC) visit in pregnant women.

**Design:**

Retrospective matched nested case-control study.

**Setting:**

Two health centres, Belén and 6 de Octubre, in the Peruvian Amazon.

**Population:**

All pregnant women who had attended ANC during the years 2010, 2011, and 2012.

**Methods:**

All cases (819 women initiating ANC in their first trimester) were selected from ANC registries from 2010 to 2012. A random sample of controls (819 women initiating ANC in their second or third trimester) was matched 1:1 to cases on health centre and date of first ANC visit. Data were obtained from ANC registries. Conditional logistic regression analyses were performed.

**Main outcome measure:**

Case-control status of each woman determined by the gestational age at first ANC visit.

**Results:**

Cases had higher odds of: 1) being married or cohabiting (aOR = 1.69; 95% CI: 1.19, 2.41); 2) completing secondary school or attending post-secondary school (aOR = 1.45; 95% CI: 1.02, 2.06); 3) living in an urban environment (aOR = 1.79; 95% CI: 1.04, 3.10) and 4) having had a previous miscarriage (aOR = 1.56; 95% CI: 1.13, 2.15), compared to controls. No statistically significant difference in odds was found for parity (aOR = 1.08; 95% CI: 0.85, 1.36).

**Conclusions:**

This study provides empirical evidence of determinants of first ANC attendance. These findings are crucial to the planning and timing of local interventions, like deworming, aimed at pregnant women so that they can access and benefit fully from all government-provided ANC services.

## Introduction

Antenatal Care (ANC) is universally accepted to be the most important determinant of pregnancy outcomes and is strongly associated with a reduction in maternal deaths [1]. Access to ANC is severely limited in most developing countries, and even if they do exist, utilization rates can be low [2, 3]. Determinants of low uptake include woman’s education, husband’s education, marital status, household income, woman’s employment, having a history of obstetric complications, parity and cultural beliefs, among others [3]. Several of these determinants are likely country and/or culture-specific. Culture-specific research is therefore needed to appropriately address this issue.

Belén is an impoverished community in the northern Peruvian Amazon accessible only by river or air transport. Most women in this community who attended ANC at the two health centres in this study (>98%) were insured by a national health plan through the Ministry of Health that covers those who are unable to afford healthcare. This health plan is called SIS (Seguro Integral de Salud) and was created in 2001 to expand health care coverage by eliminating user fees for basic health services [4]. No woman must pay for ANC services. Salaried Peruvians are covered by a national health plan called EsSalud (Seguro Social de Salud del Peru). The armed forces and police also have a healthcare plan. Private insurance companies and private clinics do exist, but this is not a popular option in the impoverished community of Belen.

A previous study conducted in this community found that, contrary to other studies from Africa, Asia and even other parts of Peru, the use of ANC in this region is high: up to 96% of pregnant women attend at least four visits [5]. However, data on when the women initiated ANC, or what determinants might influence the timing of ANC initiation, were lacking.

The timing of ANC initiation is important when considering the integration of a deworming intervention into ANC. Parasitic intestinal worms, and especially the soil-transmitted helminths (STH), hookworm and *Trichuris*, can exacerbate and cause anemia during pregnancy [6,7, 8]. In developing countries such as Peru, anemia is one of the main underlying causes of poor pregnancy outcomes [7]. Deworming during pregnancy is considered to be an effective way to reduce anemia and subsequently poor pregnancy outcomes, in STH-endemic areas [9]. The global strategy to control STH infections has evolved over time and now centres on the routine mass administration of deworming drugs (most notably single-dose mebendazole and albendazole) to at-risk groups including preschool-age children, school-age children, women of reproductive age (including pregnant and lactating women). In 1996, WHO recommended that deworming be administered to all pregnant women (after their first trimester) in areas with a prevalence of hookworm infection greater than 20–30% and where anemia is prevalent [9]. To date, only five countries, Madagascar, Nepal, Sri Lanka, India and Kenya, have adopted this recommendation and integrated deworming, after the first trimester, into governmental ANC services [8, 10, 11].

In order to improve the planning and provision of ANC deworming interventions, in a specific setting like Belén, it is important to understand the characteristics of the women who initiate ANC early (i.e. in the first trimester), and of the women who initiate ANC late (i.e. in the second or third trimester). Therefore, this research sought to compare the determinants of pregnant women who attended their first ANC visit in their first trimester with those who attended ANC for the first time in their second or third trimester.

## Methods

### Study location and subjects

**Fig 1:**
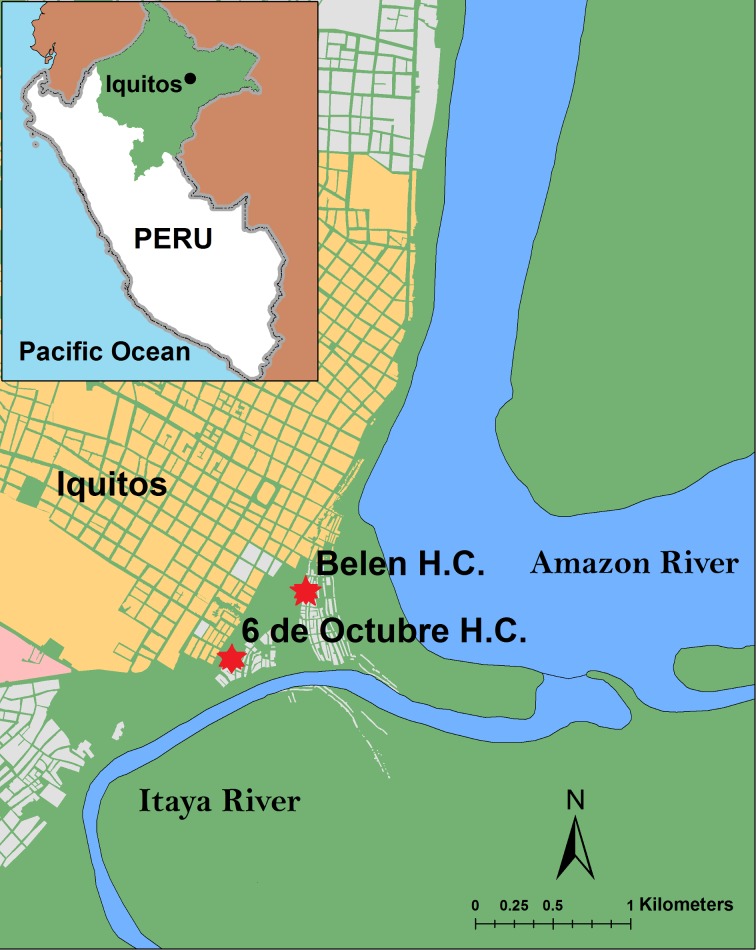
Map of study area in the northern Amazon region of Peru. Red stars show the location of the two study health centres (H.C.).

This study was conducted in the impoverished Belén district of Iquitos, the capital city of the region of Loreto, in the northeastern Amazon Jungle of Peru (Fig 1). Approximately 75,000 inhabitants live in Belén, on the floodplains of the Itaya River [12]. The *Zona Baja* (lower zone) area of Belén is prone to seasonal flooding and its population is characterized as of low socio-economic status. The Belén Health Centre and 6 de Octubre Health Centre are located in the *Zona Baja*. The source population included all pregnant women in Belén who had attended ANC at these two health centres during the years 2010, 2011, and 2012.

### Study design and sample size

A retrospective matched nested case-control study was conducted to identify determinants of early first attendance at ANC clinics (i.e. attendance in the first trimester). A case was defined as a woman whose first ANC visit was in her first trimester and a control was defined as a woman whose first ANC visit occurred in her second or third trimester. Individual matching, with a 1:1 case to control ratio, was performed. Matching factors included health centre and calendar date of first ANC visit. Exposure data were collected from three sources to ensure as little missing data as possible: ANC registries, medical charts and antenatal cards. A sample size of 789 matched sets of cases and controls was estimated to be able to detect an exposure odds ratio of 1.5 with 80% power at a significance level of 0.05 [13].

### Outcome variable

The outcome variable was the case-control status of each woman determined by the gestational age at first ANC visit. The last menstrual period (LMP) was used to determine the gestational age and trimester [9]. In instances where the LMP was missing, gestational age was based on ultrasound results; and, if this was missing, on recorded fundal height measurements taken at the first ANC visit. A gestational age of 12 weeks or less was used to define the first trimester of pregnancy [14].

### Determinants of interest

Based on the literature, parity (i.e. nulliparous vs. multiparous) was chosen as the primary determinant of interest [3]. The secondary determinants of interest included: age (i.e. early adolescent (10–14 years of age), late adolescent (15–19 years of age) or adult (≥ 20 years of age)), type of health insurance (i.e. public (SIS), private (EsSalud) or armed forces/police), education (i.e. illiterate or primary school incomplete, secondary school incomplete, or secondary school complete), marital status (i.e. single vs. living with a partner), number of previous pregnancies (gravida), number of miscarriages, number of children alive at the first visit and geographic area of residence (i.e. urban, peri-urban or rural). The urban zone included the streets located above the river floodplains. The peri-urban zone included the streets located in the zona baja. The rural zone extended from the peri-urban zone and was comprised of the communities of houses that float on the river when the water is high.

### Data collection

From May 23 to August 8, 2013 data were extracted in the health centres and entered into Microsoft Office Excel 2007 on a password-protected laptop computer.

### Assembly of the nested case-control study population

A sampling frame of all women in the ANC registries between 2010–2012 from the two health centres was generated, including each woman’s age, date of first ANC visit and gestational age at ANC initiation. From this sampling frame, all cases were identified. A second sampling frame of all eligible controls for each case (based on matching factors) was generated. Matched controls (i.e. matched on health centre and date of first visit), were randomly selected, using a computer-based random number generator, from this second sampling frame. Caliper matching was used, in the event that no exact match existed for a case, based on date of first visit. If this occurred the eligible control with the date of first visit closest to the case was selected with a two-day caliper as the aim. In the event that a woman had attended ANC for more than one pregnancy during the study period, one pregnancy was randomly selected using a computer-based random number generator.

### Missing data and data validation

Extensive measures were taken to obtain a complete dataset. These included using two additional sources of data to complement the registry data: medical charts and antenatal cards. For efficiency, for any missing data, antenatal cards were reviewed first, and then medical charts. Variables recorded in these data sources included personally identifiable information (name, age, date of birth), demographic information (education level, marital status, type of insurance), obstetric information (previous pregnancies, miscarriages, number of children alive, date of last menstrual period, expected delivery date, gestational age and ultrasound result) and clinical laboratory results (hemoglobin, hematocrit, blood type and group, glucose, syphilis and HIV results). There was a large amount of missing data (between 12% and 88%) for clinical variables and evidence that these clinical tests were not routinely conducted. Therefore, these variables were not included in analyses.

### Details of ethics approval

This study was granted ethics approval in writing by both the Research Ethics Board of the McGill University Health Centre (MUHC) in Canada and the Universidad Peruana Cayetano Heredia in Peru. Data were collected from local health centre ANC registries, medical charts, and antenatal cards and no direct contact was made with any study subject. Extracted data did not include any nominal information and unique identification numbers ensured data confidentiality.

### Statistical analyses

All analyses were performed using STATA software version 13.0 (StatCorp LP, College Station, TX, USA).

### Descriptive statistics

Descriptive statistics for all variables (frequencies, proportions, means, medians, minimums and maximums), describing baseline characteristics were calculated separately for cases and controls. The proportion of missing data for each variable was recorded. For continuous variables, the mean and standard deviation (+/- SD) was computed and for dichotomous and categorical variables, counts and proportions were computed. Significance testing of means and proportions was performed using student t-tests, and chi-square tests.

Additionally, the mean gestational age (+/- SD) was calculated, overall, by health centre, by year, and by age group. Finally, the proportion of women attending their first ANC visit by trimester, by age group and by year, was calculated.

Potential determinants of early ANC initiation had been identified *a priori* from a review of the literature. These variables, assessed at the first ANC visit, included age, geographic area of residence (urban, peri-urban, or rural), education, marital status, type of health insurance, number of previous pregnancies, parity, number of miscarriages, and number of children alive at first ANC visit. The univariate relationships among determinants and between determinants, and the outcome variable, were then summarized using two-by-two tables, bar plots, scatter plots and/or box-plots according to the most appropriate representation of the variables (continuous/dichotomous) in order to explore the relationships among variables and the possibility of confounding. Variability and collinearity were also assessed.

### Identification of determinants of early ANC initiation

Univariate conditional logistic regression was used to compute crude odds ratios (ORs) and their 95% confidence intervals (CIs). Multiple conditional logistic regression was then used to obtain adjusted odds ratios (aORs). Variables that were determined to be statistically significant in the univariate regressions and those identified from the literature were selected for the multiple conditional logistic regression model. The precision of all point estimates was assessed with 95% CIs. The primary independent exposure variable for all analyses was parity.

## Results

### Source population

A total of 2,534 pregnant women presented for a first ANC visit at either Belén Health Centre or 6 de Octubre Health Centre between January 1, 2010 and December 31, 2012.

### Study population

A total of 112 women had two pregnancies within the study period. Among the 2,534 women, 819 met the case definition. A total of 819 matched controls were randomly sampled from the sampling frame of 1,715 women who had first attended ANC in their second or third trimester. The small increase in sample size from the originally estimated 789 matched pairs was in order to include all cases from three full years (2010, 2011 and 2012).

### Results of matching cases and controls

All controls (100%) were successfully matched to cases on the first matching criteria (i.e. attending ANC at same health centre) and 82.3% on the second criteria (i.e. date of first ANC visit within two days apart of each other) (S1 Table). The average difference between a matched case-control pair’s date of first ANC visit was less than two days (1.48 (± 2.94) days). More than half (i.e. 53.5%) of the pairs were matched exactly on the date of their first ANC visit with fewer pairs matched further apart. Approximately 5% of pairs were matched more than six days apart.

### Characteristics of the study population

Cases and controls were different with respect to age, marital status, education, geographic area of residence and number of total ANC visits (Table 1). They were similar with respect to number of previous pregnancies, number of previous miscarriages, number of children alive at the first ANC visit and parity. Approximately 35% of both cases and controls were nulliparous and 65% were multiparous.

**Table 1:** Characteristics of pregnant women who attend their first 1^st^ ANC visit in the 1^st^ trimester of pregnancy (cases) and of pregnant women who first attend in the 2^nd^ or 3^rd^ trimester (controls), Belén, Iquitos, Peru, 2010–2012.

Characteristics	Cases [≤12 weeks gestation](N = 819)		Controls [>12 weeks gestation](N = 819)		Significance
	No.	%	No.	%	
Age (in years)					X^2^ = 9.5, df = 2, p = 0.008
10–14^1^	14	**1.7**	20	**2.4**	
15–19	200	**24.4**	251	**30.6**	
≥20	604	**73.7**	548	**66.9**	
Missing^2^	1	**0.1**	~	**~**	
Age (years) [mean (sd)]	25.0 (±6.78)	**Range: 12–43**	23.8 (±6.75)	**Range: 12–42**	P<0.001
Marital Status					X^2^ = 9.3, df = 1, p = 0.002
Not Married	69	**8.4**	109	**13.3**	
Married/Living together	679	**82.9**	654	**79.9**	
Missing	71	**8.7**	56	**6.8**	
Education					X^2^ = 17.2, df = 5, p = 0.004
Illiterate	6	**0.7**	6	**0.7**	
Some primary	101	**12.3**	109	**13.3**	
Primary complete	106	**12.9**	134	**16.4**	
Some Secondary	286	**34.9**	332	**40.5**	
Secondary Complete	206	**25.2**	153	**18.7**	
University or Technical College	53	**6.5**	38	**4.6**	
Missing	61	**7.5**	47	**5.7**	
Parity					X^2^ = 0.04, df = 1, p = 0.833
Nulliparous	286	**34.9**	283	**34.6**	
Multiparous	526	**64.2**	532	**65.0**	
Missing	7	**0.9**	4	**0.5**	
Geographic area of residence					X^2^ = 23.6, df = 2, p<0.001
Urban	51	**6.2**	25	**3.1**	
Peri-Urban	644	**78.6**	605	**73.9**	
Rural	124	**15.1**	189	**23.1**	
Missing	~	**~**	~	**~**	
Health Insurance					p = 0.125^3^
SIS	809	**98.8**	811	**99.0**	
EsSalud	4	**0.5**	0	**0**	
Armed Forces or Police	0	**0**	1	**0.1**	
Missing	6	**0.7**	7	**0.9**	
Number of previous pregnancies [mean (sd)]	1.71 (±1.74)	**Range: 0–10**	1.72 (±1.87)	**Range: 0–12**	p = 0.855
Number of previous miscarriages [mean (sd)]	0.24 (±0.56)	**Range: 0–3**	0.16 (±0.53)	**Range: 0–9**	p = 0.0045
Number of children alive at 1st ANC visit [mean (sd)]	1.40 (±1.48)	**Range: 0–10**	1.51 (±1.62)	**Range: 0–10**	p = 0.161
Number of total ANC visits [mean (sd)]	6.05 (±2.77)	**Range: 1–13**	4.64 (±2.24)	**Range: 1–10**	p<0.001

^1^ Youngest girl was 12 years old

^2^ The symbol ~ means zero missing data

^3^ Fisher’s exact test

In terms of health insurance, nearly all of the women were covered by SIS with the exception of five women who were either covered by EsSalud or the armed forces and police plans. Due to the low degree of variability, insurance was not included in further analyses.

In all, 90.7% of the data came from the antenatal registries, with the remaining 9.3% coming from the antenatal card and/or medical chart. The majority of cases and controls had their gestational age measured by LMP (n = 1,581; 96.4%), with the remaining from ultrasound results (n = 30; 1.8%) and fundal height (n = 27; 1.6%).

### Determinants of ANC initiation in the first trimester

The number of previous pregnancies, the number of children alive at first ANC visit, age and parity did not have statistically significant associations with being a case or control, on univariate analysis. Furthermore, number of previous pregnancies, number of children alive at first ANC visit and age, were all found to be highly correlated with parity (r = 0.95, r = 0.98, and r = 0.73, respectively). All other correlations were confirmed not at risk for collinearity.

Due to the lack of statistical significance in univariate analyses and to avoid the risk of collinearity, number of previous pregnancies, number of children alive at first ANC visit and age were removed from the multivariable analyses. Parity was selected to remain in analyses, as it was identified in the literature review as a strong determinant of ANC initiation in the first trimester. A subgroup analysis was performed to investigate the effect of parity among only adult women and also among only late adolescents in order to try to unmask the confounding effect of age. Parity remained not statistically significant in both analyses.

Additional univariate analyses found that marital status, geographic area of residence, completing secondary school or attending university or technical school, and whether or not the woman had previous miscarriage(s), all had statistically significant associations with being a case or control. Therefore, these variables were also included in the multiple regression model. The geographic area of residence variable was re-categorized. The rural and peri-urban residences were combined into one category (called peri-rural) for multivariable analyses, recognizing that the differences between rural and peri-urban residences in this study were not substantially dissimilar in terms of sanitation, electricity and water services.

In the multiple regression model, cases and controls differed significantly in geographic area of residence, marital status, education level, and previous miscarriage status (Table 2). Adjusted odds ratios (aOR) indicated that cases had higher odds of 1) being married or living with a partner (aOR = 1.70; 95% CI: 1.19, 2.41); 2) completing secondary school or attending university or technical school (aOR = 1.45; 95% CI: 1.02, 2.06); 3) living in an urban environment (aOR = 1.79; 95% CI: 1.04, 3.10); and 4) having had a previous miscarriage (aOR = 1.56; 95% CI: 1.13, 2.15), compared to controls.

**Table 2:** Unadjusted and Adjusted Odds ratios^1^ for determinants of early first ANC attendance in the Belén community of Iquitos, Peru, 2010–2012.

Variable	Univariate		Multiple regression	
	Unadjusted Odds ratio	95% Confidence Interval	Adjusted Odds Ratio	95% Confidence Interval
Parity				
Nulliparous	**REF**		**REF**	
Multiparous	**1.02**	0.83, 1.24	**1.08**	0.85, 1.36
Marital Status				
Not married	**REF**		**REF**	
Married/Living together	**1.67**	1.19, 2.34	**1.69**	1.19, 2.41
Education				
Illiterate and some primary	**REF**		**REF**	
Primary complete and some secondary	**0.94**	0.70, 1.28	**0.90**	0.66, 1.23
Secondary Complete, University or Technical College	**1.54**	1.11, 2.15	**1.45**	1.02, 2.06
Geographic area of residence				
Peri-Rural	**REF**		**REF**	
Urban	**2.24**	1.34, 3.74	**1.79**	1.04, 3.10
Number of previous miscarriages				
No previous miscarriage	**REF**		**REF**	
Previous miscarriages (1+)	**1.64**	1.22, 2.18	**1.56**	1.13, 2.15

^1^ Conditional logistic regression was used for both univariate and multivariable analyses.

### Frequency of ANC visits among cases and controls

It was found that in the nested case-control study population (N = 1,638), the total number of ANC visits that women (cases and controls) attended during their pregnancies ranged from one to thirteen, with a median of six. Among cases, the median was six and among controls the median was five. The majority of women (88%) attended more than one visit. There were 1,206 women (74%) who met WHO’s ANC recommendation of at least four visits while 905 women (55%) attended at least six ANC visits in accordance with the Peruvian Ministry of Health (MOH) recommendation.

### Descriptive statistics of the source population

#### Mean gestational age of source population at first ANC visit

The mean gestational age of all the women presenting for a first ANC visit, including all of the pregnancies from the 111 women who had two pregnancies and the one woman who had three, in the study period between January 1, 2010 and December 31, 2012 at either health centre (N = 2,647 pregnancies), was 17.4 (± 7.94) weeks. The earliest that a woman came for her first ANC visit was at four weeks and the latest was at 40 weeks. Adult women attended earlier than early or late adolescents. Among early adolescents, the mean gestational age at the first ANC visit was 18.0 ± 7.06 weeks. Late adolescents had a mean gestational age of 18.1 ± 7.76 weeks at the first ANC visit, and adult women had a mean gestational age at the first ANC visit of 17.1 (± 8.02 weeks).

#### Proportions of all pregnancies, at first ANC visit, by trimester

**Fig 2:**
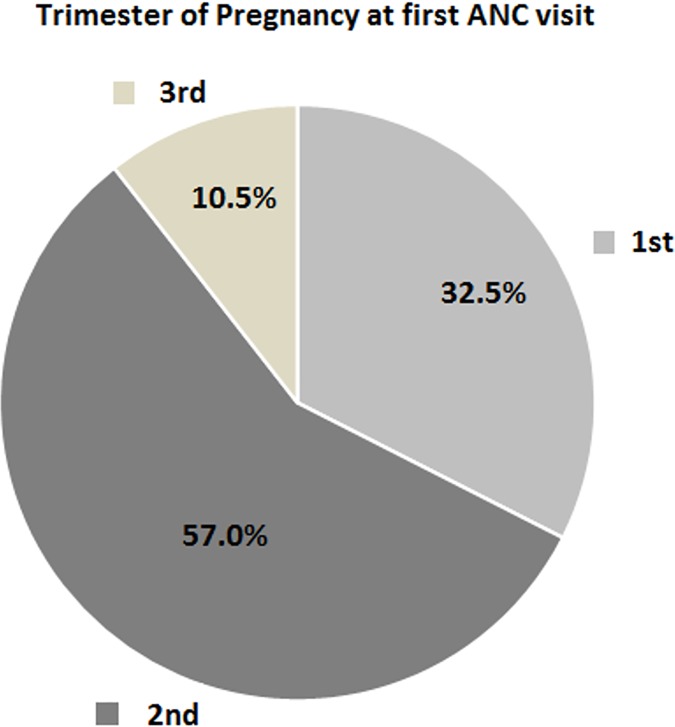
Proportions of pregnancies of women attending their first ANC visit in each of the three trimesters of pregnancy in the community of Belén, Iquitos, Peru 2010–2012.

Among all 2,647 pregnancies, 860 (32.5%) were in the first trimester, 1,508 (57.0%) were in the second trimester, and 279 (10.5%) were in the third trimester (Fig 2).

#### Proportions of all pregnancies, at first ANC visit, by trimester and age

At the first ANC visit, there were more pregnancies of adult women who were in the first trimester, compared to both early and late adolescents (35.0% versus 27.5% and 26.8%, respectively) (S1 Fig).

#### Proportions of all pregnancies, at first ANC visit, by trimester and year at the two health centres

There were more pregnancies at first ANC visit in their first trimester in 2012 at both health centres when compared to the other two years. However, there were fewer pregnancies in 2012 compared to 2010 and 2011 (S2 Table).

## Discussion

### Main findings

#### ANC attendance in Belén, in Peru and globally

An increase in the number of women attending ANC around the globe, especially in Latin America, since 2000, has been observed; however, there are little data to show that more women are attending their first visit in the first trimester as recommended by WHO [2,15]. The Peruvian national median gestational age at the first ANC visit is estimated at 2.9 months [15]. In the remote Belén community, it is 4 months. This disparity could be due to the remoteness of the Amazon region and the high levels of poverty and local beliefs and customs that prevent seeking of conventional healthcare services [16]. Local beliefs include the perception that traditional healers and alternative medicine are more effective than conventional healthcare services, especially when it can take upwards of 4 hours by boat to reach a conventional health centre when the traditional healer is located within the community. Other local perceptions that prevent women from seeking ANC may include the belief that it is shameful to have many children, or that the ANC wait time is long and the hours of operation at the health centres are inconvenient. Some local customs may also prevent a woman from receiving care from a male health worker [17].

#### Summary of determinants of early first ANC attendance

Marital status, education level, location of residence, and having had a previous miscarriage were found to be significantly associated with attending ANC in the first trimester. These findings are consistent with previous research conducted in underserved populations in developing countries [3, 18]. Marital or cohabitating partners may provide support to attend ANC earlier [18]. Data on women’s employment was not available, but located near one of the two health centers where data were collected for this study, is the large Belen community market. Many of the stalls are run by women and in conversations with health care center staff, one of the major barriers to attending ANC is leaving the market stall for a day unopened or unattended. Married or cohabitating women may have more support from their partners to keep the stall open while she is attending ANC. More years of schooling may promote autonomy, increased knowledge of ANC benefits and greater decision-making power [3, 19, 20]. Living closer to a health centre may promote ANC use as costs incurred for transportation and absence from work might be minimized [2, 3]. Women with a previous miscarriage may better understand the risks of pregnancy [21].

Contrary to much of the published literature, we found no evidence that age or parity delayed ANC initiation to the second or third trimester. If adolescent women, who have low parity, are less likely to seek ANC early in their pregnancies just as older women, who have high parity, are also more unlikely to seek ANC early, then this may also explain the lack of an association between parity and timing of first ANC visit [22]. Further investigation into the dynamics of the relationships between maternal age, parity and timing of first ANC visit is necessary.

#### Application of findings to antenatal deworming

Previous research has demonstrated that the prevalences of hookworm infection and anemia, are much greater than 20% among pregnant women in the study area [23]. Therefore, WHO guidelines would warrant an antenatal deworming intervention in this community.

ANC is an opportune time for a deworming intervention in this community since many women attend, and the benefits of treatment are great. Recently, the regional government of Loreto has initiated a deworming program offered to women after 35 weeks of pregnancy. The results of our study provide additional evidence to encourage health authorities to offer deworming earlier in pregnancy in order to more effectively reduce anemia and other worm-related morbidity during pregnancy, while avoiding inadvertent deworming in the first trimester. Ideally, for an optimal deworming intervention, women should attend their first ANC visit in their first trimester so that they receive education about worm infections and anemia, with the deworming treatment then administered at the very beginning of the second trimester. Health promotion activities directed to encouraging first time ANC attendance in the first trimester of pregnancy therefore should be encouraged.

The determinants of the timing of the first ANC visit identified in this study can provide useful information to health authorities in planning these health promotion activities. Additional efforts might be considered to ensure that especially at-risk pregnant women are reached.

### Strengths and limitations

There are several strengths of this research. The sample size was large and there was sufficient power for the analysis. The study included all women who attended their first ANC visit in the first trimester as cases and a random sample of all eligible controls. This avoided potential selection bias. It also increases the generalizability of these results to all mothers within the catchment area of Belén Health Centre and 6 de Octubre Health Centre. Using additional years of data, from 2010 to 2012, also helped to make these results more generalizable as it avoided any period effect associated with any one of the three years of data that could make one year unusual.

Limitations of this study pertain to the study design and the available data that were collected. The disadvantages of the case-control study design are well known [24]. Some issues encountered in this study included the validation of data collected, which was difficult, if not impossible, and the lack of control for extraneous variables that were not collected. The data collection required manual abstraction and was limited to certain recorded variables. For example, no data were recorded on the husband’s education or employment, the woman’s employment, the woman’s age at first birth, the desirability of the pregnancy, the household wealth, the travel time to the clinic, ethnicity, religion, or the woman’s autonomy. These variables are included in many studies in the literature but it was not possible to investigate them in this study.

Additionally, due to reliance on routinely collected data recorded in ANC registries, only information on women who had attended ANC was collected. Therefore, it was not possible to assess or identify characteristics of ANC non-attendees and these women were excluded from the study, which may have introduced some selection bias. However, based on local expert input and past experience, it is likely that these would be few, given the high proportion of all women who attend ANC in Iquitos and in Peru.

As with most epidemiologic studies there were missing data. In this case, two variables (marital status and education level) had missing data; however, the amount of missing data in each individual variable did not exceed 9% in any instance. Therefore, it is unlikely that the missing data would have biased the results of this study.

Lastly, there is potential for an information bias with regard to the date of a woman’s last menstrual period (LMP). However, in areas where ultrasound (the gold standard to estimate gestational age) is not possible for every woman, such as in Belén, WHO recommends using LMP and considers it to be a valid measure [14].

### Areas of future research

Further research into evaluating the spectrum of ANC care from first attendance to delivery in terms of availability, access, content and quality would contribute a comprehensive evidence-based approach to this critical type of health service. It would also be important to conduct further research to understand determinants of not attending ANC at all, and to include cultural and psychosocial variables to help identify real and perceived barriers. Lastly, qualitative research would be beneficial for exploring women’s satisfaction, autonomy and decision-making processes in relation to ANC.

## Conclusion

Historically, there has been a lack of local data to inform intervention activities in this region of Peru. The results of this case-control study contribute new data to this recognized research gap in the attendance and timing of antenatal care visits. These findings are crucial to the planning and timing of local interventions, like deworming, which, ultimately, also contribute to improving maternal and child health in Peru.

## Supporting information

S1 Table Results of matching controls to cases on health centre and date of first ANC visit, Belén, Iquitos, Peru, 2010–2012. (DOCX)

S2 Table Proportions of all pregnancies of women attending their first ANC visit, by trimester, at Belén Health Centre and 6 de Octubre Health Centre, Iquitos, Peru, 2010–2012. (DOCX)

S1 Fig Proportions of pregnancies of early adolescent, late adolescent and adult women, by trimester at first ANC visit, in the community of Belén, Iquitos, Peru, 2010 to 2012. (TIF)

S1 File Case Control Final Dataset. (XLS)

S2 File Codebook for Case Control Final Dataset. (XLSX)
