# Supplementary material for: Determinants of first trimester attendance at antenatal care clinics in the Amazon region of Peru: A case-control study
Source: PLoS One. 2017 Feb 16;12(2):e0171136. doi: 10.1371/journal.pone.0171136 (PMC5313205; doi:10.1371/journal.pone.0171136)
Supplement: S1 Table — (DOCX) [file pone.0171136.s001.docx]

Table S1. Results of matching controls to cases on health centre and date of first ANC visit, Belén, Iquitos, Peru, 2010-2012.

| **Matching Variables** | **Belén** | **6 de Octubre** | **Health Centres Combined** |
| --- | --- | --- | --- |
| **Health Centre** |  |  |  |
| Number of matched pairs | 380 | 439 | **819** |
| % of matched pairs from same health centre | 100% | 100% |  |
| % of total sample | 46.4% | 53.6% | **100%** |
| **Date of 1^st^ ANC visit** |  |  |  |
| Number (%) of pairs matched within 2 days | 329 (86.6%) | 345 (78.6%) | **674**  **(82.3%)** |
| Number (%) of pairs matched >2 but ≤6 days | 40 (10.5%) | 66 (15.0%) | **106**  **(12.9%)** |
| Number (%) of pairs matched > 6 days | 11  (2.9%) | 28  (6.4%) | **39**  **(4.8%)** |
