# Supplementary material for: Determinants of first trimester attendance at antenatal care clinics in the Amazon region of Peru: A case-control study
Source: PLoS One. 2017 Feb 16;12(2):e0171136. doi: 10.1371/journal.pone.0171136 (PMC5313205; doi:10.1371/journal.pone.0171136)
Supplement: S2 Table — (DOCX) [file pone.0171136.s002.docx]

**Table S2.** **Proportions of all pregnancies of women attending their first ANC visit, by trimester, at Belén Health Centre and 6 de Octubre Health Centre, Iquitos, Peru, 2010-2012.**

| **Year** | **2010 (%)** | **2011**  **(%)** | **2012**  **(%)** | **Total (%)** |
| --- | --- | --- | --- | --- |
| **Both Health Centres** |  |  |  |  |
| First trimester | 282 (30.4) | 287 (30.1) | 291 (38.1) | **860 (32.5)** |
| Second/Third trimester | 647 (69.6) | 667 (69.9) | 473 (61.9) | **1787 (67.5)** |
| Total | 929 (100) | 954 (100) | 763 (100) | **2647 (100)** |
| **Belén** |  |  |  |  |
| First trimester | 137 (27.5) | 117 (23.7) | 153 (39.0) | 407 (29.4) |
| Second/Third trimester | 362 (72.5) | 377 (76.3) | 239 (61.0) | 978 (70.6) |
| Total | 499 (100) | 494 (100) | 392 (100) | 1385 (100) |
| **6 de Octubre** |  |  |  |  |
| First trimester | 145 (33.7) | 170 (37.0) | 138 (37.1) | 453 (35.9) |
| Second/Third trimester | 285 (66.3) | 290 (63.0) | 234 (62.9) | 809 (64.1) |
| Total | 430 (100) | 460 (100) | 372 (100) | 1262 (100) |
