# Supplementary figures and images for: Determinants of first trimester attendance at antenatal care clinics in the Amazon region of Peru: A case-control study
Source: PLoS One. 2017 Feb 16;12(2):e0171136. doi: 10.1371/journal.pone.0171136 (PMC5313205; doi:10.1371/journal.pone.0171136)

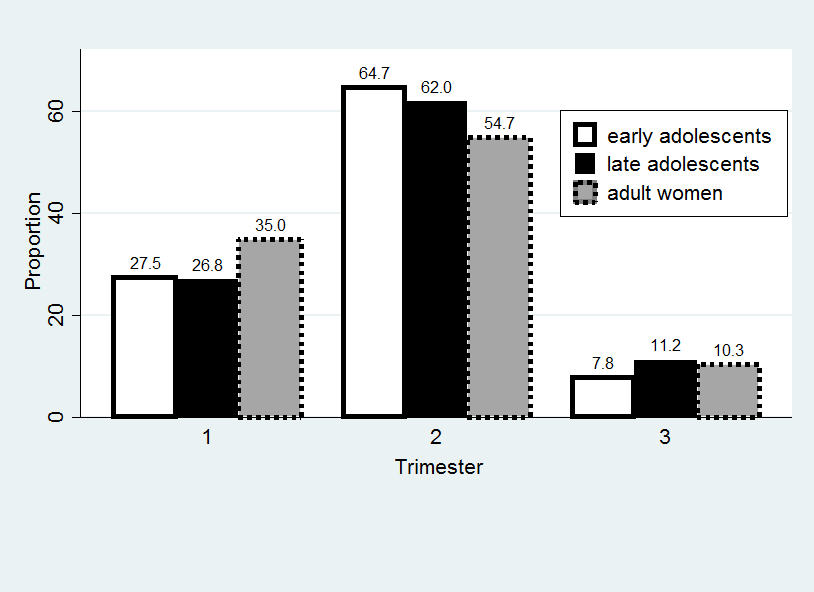

Supplement: S1 Fig — (TIF) [file pone.0171136.s003.tif]
